# Assessing drinking water quality based on water quality indices, human health risk, and burden of disease attributable to heavy metals in rural communities of Yazd County, Iran, 2015–2021

**DOI:** 10.1016/j.heliyon.2024.e33984

**Published:** 2024-07-02

**Authors:** Reza Saeedi, Sepideh Sadeghi, Mohamadreza Massoudinejad, Maryam Oroskhan, Azita Mohagheghian, Mohamadreza Mohebbi, Mehrnoosh Abtahi

**Affiliations:** a Research Institute for Health Sciences and Environment, Shahid Beheshti University of Medical Sciences, Tehran, Iran; b Department of Health, Safety, and Environment (HSE), School of Public Health and Safety, Shahid Beheshti University of Medical Sciences, Tehran, Iran; c Department of Environmental Health Engineering, School of Public Health and Safety, Shahid Beheshti University of Medical Sciences, Tehran, Iran; d MPH Department, School of Public Health and Safety, Shahid Beheshti University of Medical Sciences, Tehran, Iran; e Department of Environmental Health, School of Health, Guilan University of Medical Sciences, Rasht, Iran; f Department of Civil Engineering, Faculty of Engineering, University of Ottawa, Ottawa, Canada; g Environmental and Occupational Hazards Control Research Center, Research Institute for Health Sciences and Environment, Shahid Beheshti University of Medical Sciences, Tehran, Iran

**Keywords:** Disability-adjusted life year, Hazard quotient, Heavy metal pollution index, Incremental lifetime cancer risk, Water quality index

## Abstract

The water quality indices, health risk, and burden of disease attributable to heavy metals in rural communities of Yazd County, Iran during 2015–2021 were studied. The drinking water quality index (DWQI) based on 27 parameters (including heavy metals) and heavy metal pollution index (HPI) were used for assessing drinking water quality. The health risk and burden of disease from heavy metals in drinking water were estimated in terms of hazard quotient (HQ), hazard index (HI), incremental lifetime cancer risk (ILCR), and disability-adjusted life year (DALY). Based on the DWQI scores by community, the drinking water quality in rural communities of Yazd County was characterized as good for 61 %, fair for 25 %, marginal for 2 %, and poor for 12 %. The distribution of the rural communities into the HPI categories was as follows: 43 % for excellent, 36 % for good, 14 % for poor, and 7 % for unsuitable. In about 20 % of the rural communities, the average HI level of heavy metals was higher than the boundary limit of one. The highest average HQ level at the county level was related to arsenic (As) to be 0.44. In all the communities, the total ILCR values of the heavy metals were in the category of significant increased cancer risk (10^−6^ to 10^−4^). At the county level, As and cadmium (Cd) exhibited the two highest cancer risk levels to be 1.96 × 10^−4^ and 1.87 × 10^−4^ for ILCR, respectively. The DALY rate (per 100,000 people) induced by exposure to the heavy metals via drinking water was 13.9, which was considered relatively high as compared to that of other drinking water pollutants obtained in the previous studies. The drinking water quality improvement through decreasing Cd and As levels below the standard values can drastically reduce the attributable burden of disease and promote the public health in the rural communities.

## Highlights

• The drinking water quality in rural areas of Yazd County ranged from good (61 %) to poor (12 %).
• The HI level of the heavy metals ranged from 0.3 to 2.1 with an average value of 0.8.
• The total ILCR of the heavy metals indicated a significant increase in cancer risk.
• The DALY rate of the heavy metals was 13.9, which was considered relatively high.
• Decreasing Cd and As levels in drinking water in hotspot villages is recommended.

## 1 Introduction

Water is the source of life, a Persian proverb, shows the importance of water since ancient times. Water shortage is one of the main environmental issues for many countries, including Iran, getting more challenging by global warming and population growth. Reports show that 1.8 billion people in the world will live in water-scarce conditions by the end of 2025 [[1], [2], [3]]. Both water quantity and quality of water resources are affected by climate changes and intense human activities especially in arid and semi-arid area [4].

In rural communities and small towns, groundwater is the most reliable and feasible source of water supply. The chemical composition of groundwater is influenced by geological structures, hydrogeological conditions, and the hydro-chemical evolution of water. These factors include both natural processes and human activities, such as mining, metal and chemical industries, agricultural practices, and the application of fertilizers and pesticides [5,6]. Chemical constituents of drinking water exhibit dual beneficial and harmful health effects. Some chemicals, such as chromium, copper, and fluoride are necessary for the body in small amounts but can cause adverse health effects at high levels. In contrast, some other minerals in drinking water, like cadmium (Cd), lead (Pb), and mercury (Hg) can only lead to negative health effects [[7], [8], [9]]. Globally, heavy metals, fluoride, nitrate, and microbial agents are the most important contaminants of drinking water in rural communities [[10], [11], [12]].

Several scientific approaches are available to assess the drinking water quality, including set pair analysis, matter element extension analysis, water quality index (WQI), human health risk assessment, and assessing attributable burden of disease [11,13,14]. Among these methods, the Canadian water quality index (CWQI) holds significant importance as a widely used and easily understandable tool for estimating the overall quality of drinking water [15,16]. A modified version of CWQI as drinking water quality index (DWQI) incorporates multiple water quality parameters by assigning weights for input parameters and index factors, resulting in an index score that can be easily comprehended by the general public and policy-makers. The DWQI is based on comparing the observed levels of water quality parameters with relevant standard or guideline values [13,[16], [17], [18]]. The other composite index of water quality is heavy metal pollution index (HPI) to assess the collective impact of different heavy metals on water quality [19,20]. The adverse health effects of heavy metals vary based on the exposure route, dose, and bioaccumulation, leading to a broad spectrum of acute and chronic (non-cancer and cancer) outcomes. Extensive toxicological and epidemiological studies have been conducted to identify the mechanisms, types, and amounts of the health effects of heavy metals [[21], [22], [23]]. Along with applying water quality index, assessing the human health risk and burden of disease from exposure to drinking water contaminants exhibit potential health effects of drinking water pollutants for evaluating the performance of national and regional drinking water supply systems [20,24,25]. The simultaneous assessment of state indicators (such as DWQI and HPI) and effect measures (such as human health risk and burden of disease) of drinking water quality can provide deeper insights into the drinking water quality for selecting and implementing strategies of water quality management. Despite its potential benefits, this approach has not received much attention in the previous studies.

Yazd County is located in central areas of Iran. The main source of community water supply in the rural areas of the county is groundwater. In recent years, the groundwater quality in Yazd County has been threatened by several factors, mainly drought, climate change, over water withdrawal, and increasing pollution sources. Despite the high potential of degrading drinking water quality, no comprehensive assessment of drinking water quality and related health consequences in the rural communities of Yazd County has been conducted so far; therefore, the objective of this study was to analyze drinking water quality in the rural communities of Yazd County during 2015–2021 based on DWQI, HPI, human health risk, and burden of disease. The results of this study provided valuable information for health agencies and managers to develop the necessary strategies for promoting public health and reducing the environmental burden of disease. The results of this study provided valuable information for health agencies and managers to develop the necessary strategies for promote public health and reducing the environmental burden of disease.

## 2 Materials and methods

### 2.1 Study area and data collection

**Fig. 1:**
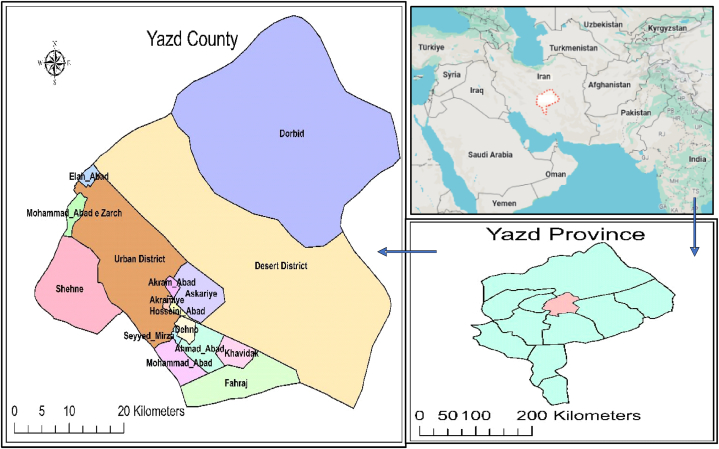
Geographic location of study area, Yazd County, Yazd province, Iran.

Fig. 1 shows the geographic location of the study area. Yazd County is in Yazd province, Iran, situated on the central Iranian plateau with an area of 2491 km^2^. The county is located between 54° 8′ to 54° 51′ east longitude and 31° 39′ to 32° 13′ north latitude. Yazd County experiences a dry and hot summer with low humidity and minimal rainfall. Based on the 2016 census, Yazd County has a population of approximately 656,000, with around 5.6 % (approximately 37,000 person) residing in rural communities. The main factors threatening water quality in Yazd Province include industrial activities, urbanization, agricultural practices, climate changes, mining activities, and the discharge of untreated municipal and industrial wastewater into the water bodies [[26], [27], [28], [29]]. The main source of community water supply in the county’ rural communities is groundwater. The water treatment plants in the rural communities only consist of chlorination for control of microbial contamination.

This study was conducted in collaboration with the Department of Health Affairs at Yazd University of Medical Sciences. The Department of Health Affairs provided the data of drinking water quality examinations in 14 villages in Yazd County during 2015–2021 (Mohammadabad, Shehneh, Fahraj, Ahmadabad, Khavidak, Akramabad, Seyyed Mirza, Dehno, Askarieh, Hosseinabad, Akramieh, Mohammadabad Zarach, Dorbid, and Allahabad) with a total population of 36,695. The population of the rural areas in Yazd County is documented in Table S1 of supplementary materials. The examined drinking water quality parameters were pH, electrical conductivity (EC), turbidity, total dissolved solids (TDS), total hardness (TH), total alkalinity (Alk_t_), aluminum (Al), arsenic (As), calcium (Ca), cadmium (Cd), chloride (Cl), chromium (Cr), copper (Cu), fluoride (F), iron (Fe), mercury (Hg), potassium (K), magnesium (Mg), manganese (Mn), ammonia (NH_3_), nickel (Ni), nitrite (${\text{NO}}_{2}^{-}$), nitrate (${\text{NO}}_{3}^{-}$), lead (Pb), phosphate (${\text{PO}}_{4}^{3-}$), sulfate (${\text{SO}}_{4}^{2-}$), and zinc (Zn). The input drinking water quality measurements consisted of about 11,000 data records. The drinking water quality measurements were conducted in accordance with the instructions of Standard Methods [30]. To ensure data accuracy and consistency, the provided data of drinking water quality were investigated and cleaned by removing outliers by the methods applied by Gholamnia et al. [31].

### 2.2 Calculation of the DWQI

In this study, the DWQI developed by Abtahi et al. [17] as a modified version of the CWQI was used. The DWQI is calculated based on annual water quality measurements using three factors to be scope factor (F_1_), frequency factor (F_2_), and amplitude factor (F_3_). The F_1_ is a function of the number of parameters violating the standard range. The F_2_ is representative of the proportion of the violator measurements. The water quality parameters included in the DWQI and their standard values and weights are provided in Table S2. F_1_ and F_2_ were calculated using equations (1), (2)) [17]:

(1) $$F_{1}=\frac{\sum_{i=1}^{m}w_{i}}{\sum_{i=1}^{n}w_{i}}$$

(2) $$F_{2}=\frac{\sum_{i=1}^{m}\left(w_{i}{Mi/}_{N_{i}}\right)}{\sum_{i=1}^{n}\left(w_{i}\right)}$$

where *w*_*i*_ is the weight of the input parameter *i*, n is the number of input parameters, *m* is the number of violator parameters, *M*_*i*_ is the number of violator measurements of input parameter *i* and *N*_*i*_ is the total number of measurements of input parameter *i* [17,18]. The F_3_ was determined based on the excursion levels of input parameters and the normalized sum of excursions using equations (3), (4), (5), (6), (7), (8)) [17]:

(3) $$E_{i}=\frac{\sum_{N_{i}}\frac{C_{i}}{{SV}_{i}}}{N_{i}}\;for\;bioaccumulative\;or\;\text{carcinogen}\;parameters$$

(4) $$E_{i}^{\prime }=\frac{\sum_{M_{i}^{\prime }}10^{\left(\left(V_{i}^{\prime }-{SV}_{i}^{\prime }\right)-1\right)}}{N_{i}^{\prime }}\;\text{for}\;\text{logarithmic}\;\text{scale}\;\text{parameters}$$

(5) $$E_{i}^{\prime \prime }=\frac{\sum_{M_{i}^{\prime \prime }}\left(\frac{C_{i}^{\prime \prime }}{{SV}_{i}^{\prime \prime }}-1\right)}{N_{i}^{\prime \prime }}\;\text{for}\;\text{other}\;\text{parameters}$$

(6) $$NSE=\frac{\sum_{i=1}^{n}\left(W_{i}E_{i}+w_{i}^{\prime }E_{i}^{\prime }+w_{i}^{\prime \prime }E_{i}^{\prime \prime }\right)}{\sum_{i=1}^{n}w_{i},w_{i}^{\prime },w_{i}^{\prime \prime }}$$

(7) $$F_{3}=\frac{NSE}{0.01NSE+0.01}$$

(8) $$DWQI=100-\left(\frac{w_{F_{1}}F_{1}^{2}+w_{F_{2}}F_{2}^{2}+w_{F_{3}}F_{3}^{2}}{w_{F_{1}}+}\right)$$

where *Ei* is the excursion amount of bioaccumulative or carcinogen parameter *i* (including As, Cd, Cr, Hg, Ni, and Pb), $E_{i}^{\prime }$ is the excursion amount of logarithmic scale parameter *i* (in this study, only pH), $E_{i}^{\prime \prime }$ is the excursion amount of water quality parameter *i,* excluding bioaccumulative and logarithmic scale parameters, *Ci* and *SVi* are the concentration and standard value of i_th_ bioaccumulative or carcinogen water quality parameter, respectively, $V_{i}^{\prime }$ and ${SV}_{i}^{\prime }$ refer to the pH value and pH standard boundary limit, respectively, $C_{i}^{\prime \prime }$ and ${SV}_{i}^{\prime \prime }$ are respectively the concentration and standard value of i_th_ water quality parameter, excluding bioaccumulative and logarithmic scale parameters, NSE is the normalized sum of excursions of input parameters, and $w_{F_{1}}$, $w_{F_{2}}$, and $w_{F_{3}}$ are respectively the weights of F_1_ (0.385), F_2_ (0.327), and F_3_ (0.288) [17].

For calculating the DWQI in this study, we included sampling stations that measured at least seven input parameters a minimum of four times per year, which was considered as a precondition entrance to calculation. All the rural communities in Yazd County met the criteria for calculating the DWQI. To provide a comprehensive understanding of the drinking water quality, the health-based water quality index (HWQI) and acceptability water quality index (AWQI) were also determined using above-mentioned procedure and equations with relevant water quality parameters (parameters causing adverse health effects such as As and nitrate for HWQI and parameters affecting aesthetic aspects of drinking water like hardness and TDS for AWQI). The DWQI values are classified into five categories for qualitative description of drinking water quality as follows: 95–100 as excellent, 85–94.9 as good, 70–84.9 as fair, 55–69.9 as marginal, lower than 55 as poor [17].

### 2.3 Calculation of the HPI

The HPI is a quantitative measure for evaluating water quality regarding heavy metals. In the HPI, the input heavy metals are weighted and both maximum desirable and permissible levels are applied. The HPI was calculated using equations (9), (10)) [20,24,25]:

(9) $$Q_{hm_{i}}=\frac{\sum_{O_{i}}\frac{C_{{hm}_{i}}-{DV}_{{hm}_{i}}}{{SV}_{{hm}_{i}}-{DV}_{{hm}_{i}}}}{O_{i}}\times 100$$

(10) $$HPI=\frac{\sum_{i=1}^{o}w_{{hm}_{i}}Q_{{hm}_{i}}}{\sum_{i=1}^{o}w_{{hm}_{i}}}$$

where $Q_{hm_{i}}$ represents the sub-index value of heavy metal *i*, *O*_*i*_ is the number of measurements of heavy metal *i*, $C_{{hm}_{i}}$ is the concentration of heavy metal *i*, ${DV}_{{hm}_{i}}$ is the desirable concentration of heavy metal *i* in drinking water (zero for all the input heavy metals in this study), ${SV}_{{hm}_{i}}$ is the standard value of heavy metal *i* in drinking water (provided in Table S2), *o* in the number of heavy metals included in the HPI, and $w_{{hm}_{i}}$ is the weight of heavy metal *i* in the HPI. The HPI values are categorized into five groups to be excellent (0–25), good (26–50), poor (51–75), very poor (76–100), and unsuitable (>100) [32].

### 2.4 Health risk assessment

The human health risk assessment (HRA) is a systematic and organized approach utilized to assess the occurrence probability of health effects in a population exposed to certain health hazards [33]. In order to assess non-carcinogenic and carcinogenic health risks associated with exposure to heavy metals through drinking water, equations (11), (12), (13), (14), (15), (16), (17), (18), (19), (20) were employed [1,6,9,34,35]:

(11) $${CDI}_{hmi,ing}=\frac{C_{hmi}\times IR\times EF\times ED}{BW\times AT}$$

(12) $${CDI}_{hmi,d}=\frac{C_{hmi}\times SA\times F\times {PC}_{i}\times ST\times EF\times ED\times 10^{-3}}{BW\times AT}$$

(13) $${CDI}_{hmi}={CDI}_{hmi,ing}+{CDI}_{hmi,d}$$

(14) $${HQ}_{hmi,ing}=\frac{{CDI}_{hmi,ing}}{{RfD}_{hmi,ing}}$$

(15) $${HQ}_{hmi,d}=\frac{{CDI}_{hmi,d}}{{RfD}_{hmi,d}}$$

(16) $${HQ}_{hmi}={HQ}_{hmi,ing}+{HQ}_{hmi,d}$$

(17) $$HI=\sum_{i=1}^{o}{HQ}_{hmi}$$

(18) $${ILCR}_{hmi,ing}={CDI}_{hmi,ing}\times {SF}_{hmi,ing}$$

(19) $${ILCR}_{hmi,d}={CDI}_{hmi,d}\times {SF}_{hmi,d}$$

(20) $${ILCR}_{hmi}={ILCR}_{hmi,ing}+{ILCR}_{hmi,d}$$

where ${CDI}_{hmi,ing}$, ${CDI}_{hmi,d}$, and ${CDI}_{hmi}$ are the chronic daily intakes of heavy metal *i* through ingestion, dermal absorption, and both the pathways (mg/kg.d), respectively, *C*_*hmi*_ is the heavy metal *i* concentration in drinking water (mg/L), *IR* is the drinking water ingestion rate (L/d), EF is the exposure frequency (d/y), ED is the exposure duration (y), *BW* is the body weight of the target population (kg), AT is the averaging time (d), *F* is a fraction of surface skin in contact with water (dimensionless), *SA* is the skin area (cm^2^), *PCi* is the skin permeability constant of heavy metal *i* (cm/h), ST is the shower time (h/d), *HQ*_*hmi,ing*_, *HQ*_*hmi*,*d*_, and *HQ*_*hmi*_ are respectively the hazard quotient of heavy metal *i* through ingestion, dermal absorption, and both the pathways (dimensionless), *RfD*_*hmi,ing*_ and *RfD*_*hmi,d*_ are the reference doses of the heavy metal *i* via ingestion and dermal contact, respectively (mg/kg.d), HI is the hazard index of all the heavy metals (dimensionless), *ILCR*_*hmi,ing*_, *ILCR*_*hmi*,*d*_, and *ILCR*_*hmi*_ are respectively the incremental lifetime cancer risk of heavy metal *i* through ingestion, dermal absorption, and both the pathways (dimensionless), and *SF*_*hmi,ing*_ and *SF*_*hmi,d*_ are the slope factors of the heavy metal *i* via ingestion and dermal contact, respectively (kg.d/mg) [6,36]. The exposure factor amounts and RfD and SF values for the HRA are given in Table S3. The target heavy metals had not the RfD and SF for dermal absorption, therefore; the relevant values of ingestion route were applied for the HRA of dermal exposure to the heavy metals via drinking water [37]. The HQ and HI values lower than one are considered as acceptable levels (indicating no non-carcinogenic health risk), while HQ and HI values higher than one exhibit significant non-cancer risks. The values of ILCR lower than 10^−6^ are considered as acceptable levels (negligible increased cancer risk), the ILCR values ranged from 10^−6^ to 10^−4^ exhibit probable increased cancer risks, and ILCR values higher than 10^−4^ indicate significant increased cancer risks [36,38,39].

### 2.5 Estimation of burden of disease

The burden of disease attributable to exposure to As, Cd, Cr, and Pb via drinking water based on the carcinogenic risk was calculated in terms of disability adjusted life years (DALYs). The disease burden of the attributed cancers was estimated using a two-stage disease model by considering the following phases [1]: diagnosis and treatment phase and [2] remission to cure for cancer treatment and [1] diagnosis and treatment phase [2], remission to death [3], pre-final phase, and [4] final phase for death from cancer. The burden of disease induced by exposure to the heavy metals was calculated using equations (21), (22), (23)) [6,[40], [41], [42]] [7]:

(21) $${DALY}_{\;hmi,c}={YLL}_{\;hmi,c}+{YLD}_{\;hmi,c}$$

(22) $${YLL}_{\;hmi,c}=\frac{ILCR{\;}_{hmi}}{70}\times P\times \left(1-{SR}_{\;c}\right)\times \left(L-\left(a+{\;D}_{c,dt}+{\;D}_{c,rd+}{\;D}_{c,pt}+{\;D}_{c,t}\right)\right)$$

(23) $${YLD}_{\;hmi,c}=\frac{ILCR{\;}_{hmi,c}}{70}\times P\times \left({\;DW}_{c,dt}\times {\;D}_{c,dt}+SR{\;}_{c}\times {\;DW}_{c,rc}\times {\;D}_{c,rc}+\left(1-{SR}_{\;c}\right)\times {\;DW}_{c,rd}\times {\;D}_{c,rd}+\left(1-{SR}_{\;c}\right)\times {\;DW}_{c,pt}\times {\;D}_{c,pt}+\left(1-{SR}_{\;c}\right)\times {\;DW}_{c,t}\times {\;D}_{c,t}\right)$$

where *DALY*_*hmi,c*_, *YLL*_*hmi,c*_, and *YLD*_*hmi,c*_ are respectively the disability adjusted life years, years of life lost due to premature mortality, and years lived with disability caused by cancer *c* due to exposure to heavy metal *i* via drinking water, *P* is the population of the target rural community (person), *SR*_*c*_ is the survival rate of cancer *c* (dimensionless), *L* (y) is the standard life expectancy (86 y), *a* is the average age of the population of the rural communities (35 y), *D*_*c*_*,*_*dt*_, *D*_*c,rc*_, *D*_*c,rd*_, *D*_*c*_*,*_*pt*_, and *D*_*c*_*,*_*t*_ are respectively the durations of cancer *c* phases of diagnosis and treatment, remission to cure, remission to death, pre-final, and final, and *DW*_*c*_*,*_*dt*_, *DW*_*c,rc*_, *DW*_*c,rd*_, *DW*_*c*_*,*_*pt*_, and *DW*_*c*_*,*_*t*_ are respectively the disability weights of cancer *c* phases of diagnosis and treatment, remission to cure, remission to death, pre-final, and final. Table S4 provides duration and DW of the cancers caused by exposure to the heavy metals through drinking water [6,43].

### 2.6 Statistical analysis

The annual variations of the drinking water quality during 2015–2021 were statistically analyzed using the Mann-Kendall and Sen's slope estimator. The Mann-Kendall test analyzes the time series data for monotonic trend and Sen's slope estimator indicates slope of the time trend. The Mann-Kendall test and Sen's slope estimator are powerful non-parametric methods for analyzing trends in time series data. The Mann-Kendall test is used to assess whether there is a monotonic upward or downward trend over time without requiring the data to be normally distributed. The Sen's slope estimator is used to estimate the slope of the trend, which is particularly useful when the trend appears to be linear. The slope of temporal trends of water quality concentrations was determined using Sen's slope estimator as equation (24):

(24) $$Y_{i}=B_{i}+Q_{i}\left(T_{i}-T_{0}\right)$$

Where *Y*_*i*_ is the annual average value of the water quality parameter *i*, *B*_*i*_ is the estimated annual average value of the water quality parameter *i* in the first year, *Q*_*i*_ is the slope of annual trend of the water quality parameter *i* (a positive value exhibits an upward trend and a negative value indicates a downward trend), and *T*_*0*_ and *T* are respectively the first year and year *i* of the water quality measurements. The relationship between water quality parameters was investigated using one-way analysis of variance (ANOVA) and Pearson's correlation coefficient. The statistical significance was considered by *p* values less than 0.05 [31,44].

## 3 Result and discussion

### 3.1 Temporal trend of drinking water quality

Table 1 provides the average values of drinking water quality parameters in the rural communities of Yazd County and their temporal trends during 2015–2021. As showed in Table 1, the average levels of three water quality parameters exceeded the standard values to be TH (268 ± 10 mg/L as CaCO_3_), As (0.015 ± 0.011 mg/L), and Cd (0.017 ± 0.018 mg/L). These results are particularly concerning for arsenic and cadmium, as these parameters have detrimental health effects. The elevated levels of hardness and heavy metals in drinking water, especially in rural community supplied with groundwater resources were repeatedly reported in the literature. In the study of Rahman et al. [45], the average concentrations of Cr, Cd, and Pb in drinking water of the Meghna Ghat industrial area in Bangladesh were obtained 0.07, 0.007, and 0.18 mg/L which exceeded the drinking water standard. In a study by Triassi et al. [46] on heavy metals in groundwater in southern Italy, the order of heavy metal levels in the groundwater was determined to be Fe > Mn > Zn > Ni > As > Cu > Se > Pb > Cd.

**Table 1:** Average values of drinking water quality parameters in the rural communities of Yazd County and their temporal trends during 2015–2021.

Parameter	Unit	Mean ± SD	Q^a^	B^b^	Sig.
Al	mg/L	0.006 ± 0.003	−1.57E-03	1.06E-02	**
Alk_t_	mg/L	184 ± 16	7.47 E+00	1.66 E+02	*
Ca	mg/L	64.6 ± 2.4	−4.27E-03	1.62 E+02	**-**
Cl	mg/L	137 ± 7	−7.28E-01	1.42 E+02	**-**
EC	μS/cm	919 ± 42	−8.55E-01	9.36 E+02	**-**
Fe	mg/L	0.031 ± 0.007	−1.42E-03	3.41E-02	**-**
K	mg/L	1.35 ± 0.22	5.99E-03	1.28 E+00	**-**
Mg	mg/L	25.9 ± 2.5	2.75 E+00	1.00 E+02	**-**
NH_3_	mg/L	0.056 ± 0.034	−1.54E-02	1.03E-01	**
pH	–	7.72 ± 0.04	−3.85E-04	7.71 E+00	**-**
PO_4_	mg/L	0.730 ± 0.644	2.03E-02	3.78E-01	**-**
SO_4_	mg/L	84.0 ± 11.0	4.49 E+00	7.02 E+01	–
TDS	mg/L	660 ± 72	2.07 E+01	5.77 E+02	**-**
TH	mg/L as CaCO_3_	268 ± 10	3.31 E+00	2.60 E+02	**-**
Zn	mg/L	0.070 ± 0.015	4.55E-03	5.68E-02	**-**
As	mg/L	0.015 ± 0.011	−4.55E-03	2.82E-02	*
Cd	mg/L	0.017 ± 0.018	4.69E-04	1.00E-02	**-**
Cr	mg/L	0.009 ± 0.004	−1.52E-03	1.29E-02	**-**
Cu	mg/L	0.025 ± 0.012	−5.18E-03	3.95E-02	**
F	mg/L	0.482 ± 0.035	4.53E-01	4.53E-01	**-**
Hg	mg/L	0.0010 ± 0.0003	4.89E-06	1.45E-03	**-**
Mn	mg/L	0.032 ± 0.011	2.15E-03	2.23E-02	*
Ni	mg/L	0.005 ± 0.004	−1.14E-03	8.47E-03	–
NO_2_^−^	mg/L	0.064 ± 0.136	−2.05E-04	1.39E-02	**-**
NO_3_^−^	mg/L	12.6 ± 2.2	−1.11 E+00	1.61 E+01	*
Pb	mg/L	0.006 ± 0.002	3.13E-04	4.89E-03	**-**
Turbidity	NTU	0.18 ± 0.19	3.79E-02	1.75E-02	**-**

a *B*: the estimated average value of each water quality parameter in the first year of assessment (parameter of Sen's slope estimator).

b *Q*: the slope of annual temporal trends of each water quality parameter (parameter of Sen's slope estimator); Sig.: significance; * and ** are 95 % and 99 % confidence levels, respectively.

Based on the Mann-Kendall test and Sen's slope estimator, five drinking water quality parameters exhibited significant downward trends (*p* value < 0.05) during the study period to be Al (an annual decrease of 0.0016 mg/L), NH_3_ (an annual decrease of 0.015 mg/L), As (an annual decrease of 0.0046 mg/L), Cu (an annual decrease of 0.0052 mg/L), and ${\text{NO}}_{3}^{-}$ (an annual decrease of 1.11 mg/L). In contrast, Alk_t_ and Mn significantly increased 7.47 and 0.0022 mg/L per year (*p* value < 0.05) during 2015–2021, respectively. In general, the drinking water quality in the rural communities was considered to get better during 2015–2021, but the drinking water quality in 2021 still did not meet the Iranian standards for Cd and TH. The temporal changes in the drinking water quality were due to the variations in water quality of applied water resources, as the water treatment plants were not changed during the study period.

### 3.2 Evaluation of drinking water quality using the DWQI

**Fig. 2:**
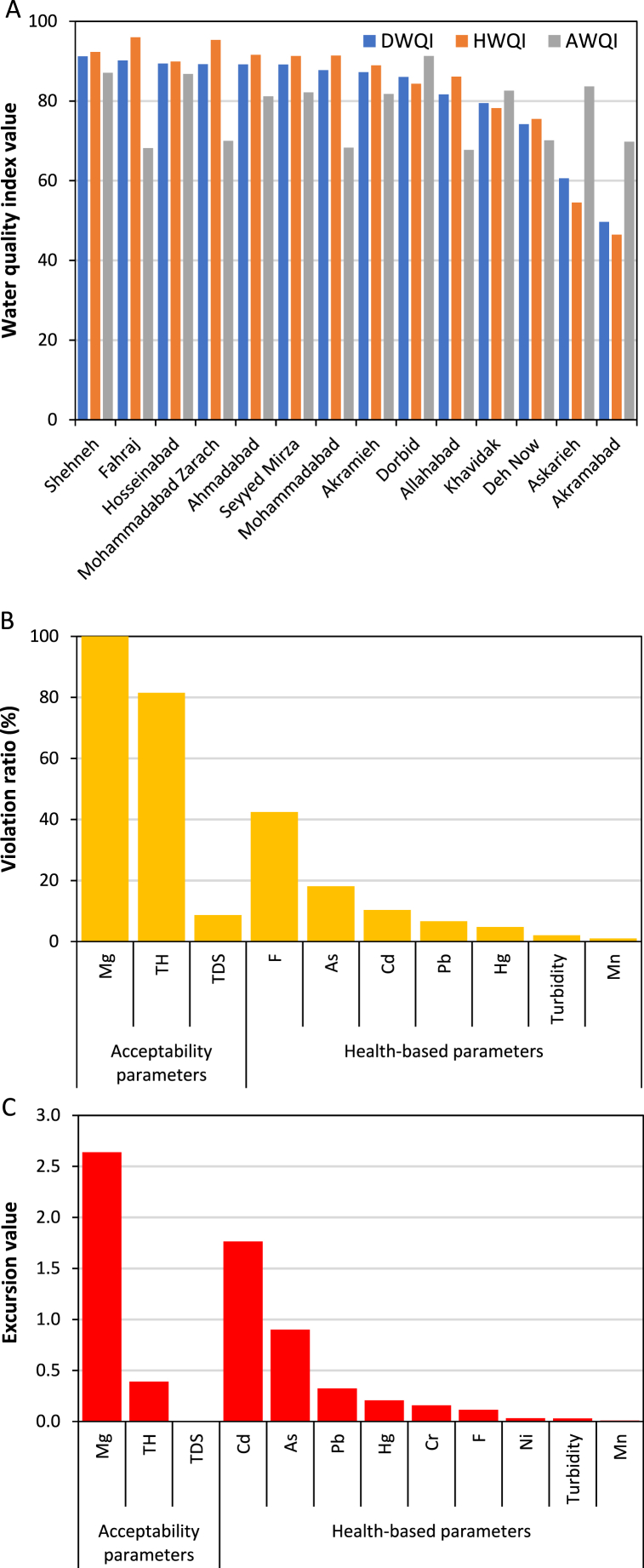
Assessing drinking water quality in the rural communities of Yazd County based on the DWQI, HWQI, and AWQI scores (A), violation ratios (B), and excursion values (C).

The comprehensive assessment of drinking water quality in the rural communities of Yazd County based on the DWQI, HWQI, and AWQI is depicted in Fig. 2(A-C). As indicated in Fig. 2(A-C), the highest average scores of the DWQI, HWQI, and AWQI by community were observed in Shehneh (91.21), Fahraj (96.00), and Dorbid (91.31), respectively. In contrast, the lowest corresponding values were related to Akramabad, Akramabad, and Fahraj to be 49.64, 46.44, and 68.15, respectively. Due to the much higher weight factor of health-based parameters (0.76 versus 0.24), the DWQI scores were close to the HWQI values, so that the overall average DWQI, HWQI, and AWQI scores of drinking water in the rural communities were determined to be 80.6, 82.3, and 75.0, respectively. Only three out of 11 acceptability water quality parameters were identified as the violator parameters to be TH (100 %), Mg (81.5 %), and TDS (8.7 %). The excursion values of the violator acceptability parameters were as follows: 2.6 for Mg, 0.4 for TH, and 0.005 for TDS. The number of violator health-based parameters were seven to be higher than that of the violator acceptability ones, but their violation percentages were relatively low (42.4 % for F, 18.1 % for As, 10.4 % for Cd, 6.7 % for Pb, 4.8 % for Hg, 2.0 % for turbidity, and 1.0 % for Mn). All the F violation cases were lower than the minimum permissible values (0.5 mg/L). The excursion values of the health-based parameters were determined to be 1.76 for Cd, 0.90 for As, 0.32 for Pb, 0.21 for Hg, 0.16 for Cr (without violating the standard value), 0.11 for F, 0.03 for Ni (without violating the standard value), 0.03 for turbidity, 0.01 for Mn.

**Fig. 3:**
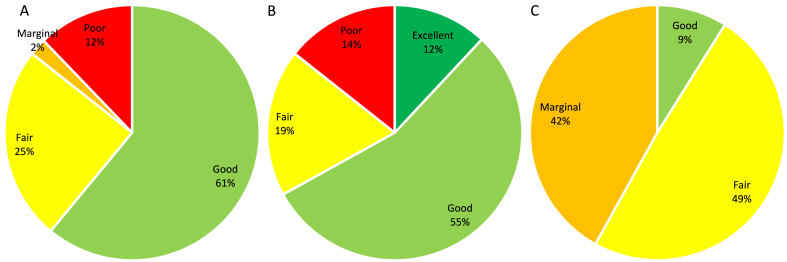
Description of drinking water quality in the rural communities of Yazd County based on the DWQI (A), HWQI (B), and AWQI (C).

Fig. 3(A-C) shows the description of drinking water quality in the rural communities of Yazd County based on the DWQI, HWQI, and AWQI. The distribution of drinking water quality into the DWQI categories was as follows: 61 % for good, 25 % for fair, 2 % for marginal, and 12 % for poor. As can be seen in Fig. 3(B and C), the variations in the health-based parameters and HWQI values (12 % as excellent, 55 % as good, 19 % as fair, and 14 % as poor) were much higher than the AWQI ones (9 % as good, 49 % as fair, and 42 % as marginal). According to the results, the most detrimental parameters of the drinking water in the rural communities were heavy metals.

Several previous studies evaluated the drinking water quality or drinking source water quality in Iran based on water quality indices. In the study of Mohebbi et al. [13] on assessing drinking source groundwater quality in Iran using a modified DWQI, the description of source groundwater quality in Iran by the DWQI was as 95 % for good, 3 % for fair, and 2 % for marginal. The three water parameters with the highest violation percentages were F (74.2 %), Mg (31.8 %), and ${\text{NO}}_{3}^{-}$ (12.6 %). Abtahi et al. [17] assessed the drinking source water quality in rural communities of Khuzestan Province, Iran by county. The average DWQI scores by county ranged from 69 ± 10 in Shadegan (as marginal) to 90 ± 5 in Izeh (as good). Based on the DWQI score, the characterization of drinking water resources was as follows: excellent (6.7 %), good (59.1 %), fair (26.2 %), marginal (7.8 %), and poor (0.1 %). The turbidity and Ryznar Index were respectively determined as the most violator health-based and acceptability parameters in the drinking water resources. Amiri et al. [47] classified the groundwater quality in Yazd province using entropy weighted water quality index (EWWQI). Based on the EWWQI score, the proportions of groundwater samples described as excellent, good, medium, poor, and extremely poor were 10.3 %, 30.0 %, 17.8 %, 7.4 %, and 34.5 %, respectively. Mousazadeh et al. [48] applied the modified National Sanitation Foundation Water Quality Index (NSFWQI) to evaluate the quality of surface and ground waters in Kardeh Dam Basin, northeast of Iran. According to the modified NSFWQI score, the water resources were characterized as good (59 %) and average (41 %) and the water quality exhibited a downward trend from northwest to southeast of the study area. In the study of Badeenejad et al. [49] on drinking source groundwater quality in Shiraz, the WQI values ranged from 40.01 to 117.38, and the groundwater resources were classified into excellent, good, and poor categories by 5.7 %, 65.7 %, and 28.6 %, respectively. In another study by Abbasnia et al. [50] on groundwater quality in Sistan and Baluchistan Province, water quality of 80 % the groundwater resources was characterized as good and 20 % of them was categorized as excellent. Khosravi et al. [51] in an investigation on groundwater quality in the Yazd-Ardakan Plain during 2000–2020 showed that the water quality of the region fell within the medium and acceptable classes based on the groundwater quality index (GQI), exhibiting a downward trend in water quality. In a study of water quality of the Abarkuh aquifer in Yazd Province based on the WQI by Goodarzi and Ansari [52], characterization of water samples was as follows: 70 % as good, 11 % as poor, 14 % as very poor, and 5 % as unsafe.

### 3.3 Evaluation of drinking water quality based on the HPI

**Fig. 4:**
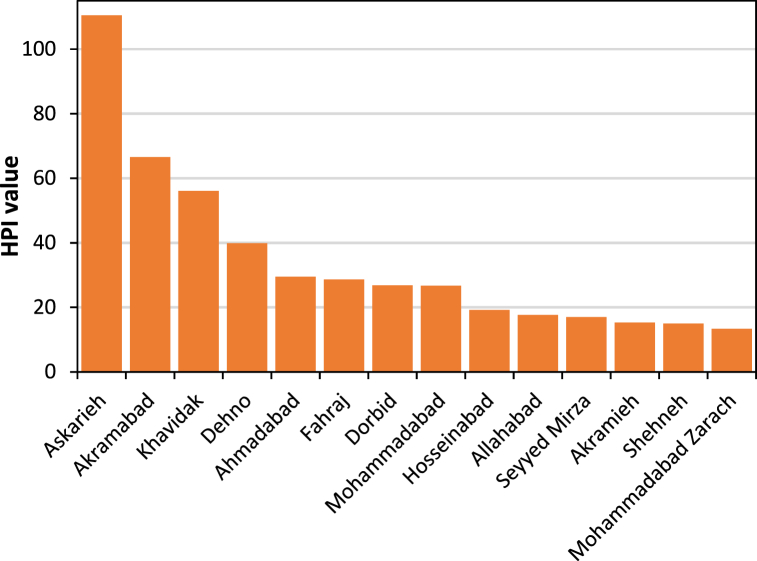
Average values of the HPI by rural community in Yazd County during 2015–2021.

Fig. 4 presents the average values of the HPI by rural community in Yazd County during 2015–2021. The average values of the HPI ranged from 13 (Mohammadabad Zarach) to 110 (Askarieh). The distribution of the rural communities into the HPI categories was as follows: 43 % for excellent (Hosseinabad, Akramieh, Allahabad, Seyyed Mirza, Shehneh, and Mohammadabad Zarach), 36 % for good (Dehno, Ahmadabad, Fahraj, Mohammadabad, and Dorbid), 14 % for poor (Akramabad and Khavidak), and 7 % for unsuitable (Askarieh). The HPI values were significantly correlated with the HWQI scores (*R*^2^ > 0.73, *p* value < 0.05), but the HWQI score was more sensitive to violation from the standard values; therefore, the HWQI was considered as a more suitable indicator to characterize drinking water quality.

In the study of Rahman et al. [45] on assessing heavy metal pollution in the groundwater of the Meghna Ghat industrial area, Bangladesh, 35 % of water samples were unsuitable (HPI>100) for drinking purposes. Abou Zakhem and Hafez [19] evaluated groundwater quality using the HPI in Damascus Oasis, Syria. The HPI values ranged from 7 to 19 with an average level of 9 which put in the excellent category.

### 3.4 Health risk assessment of heavy metals in drinking water

**Fig. 5:**
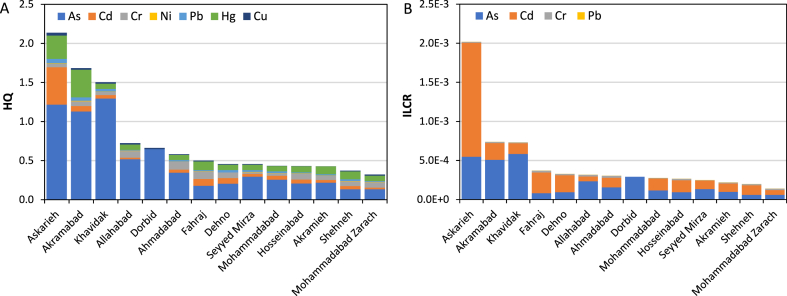
Average non-carcinogenic (A) and carcinogenic (B) risk levels of exposure to heavy metals via drinking water in rural communities of Yazd County during 2015–2021.

The average non-carcinogenic and carcinogenic risk levels of ingestion and dermal exposure to heavy metals via drinking water in rural communities of Yazd County during 2015–2021 are presented in Fig. 5(A and B). According to Fig. 5(A), As exhibited a serious non-carcinogenic health concern due to elevated HQ levels (HQ > 1) in Khavidak (1.3), Askarieh (1.2), and Akramabad (1.1). The average HQ levels of other heavy metals by community during 2015–2021 were lower than the boundary limit of 1.0. In Mohammadabad Zarach, Cd, Cr, Ni, Pb, and Hg levels in drinking water were not reported and the HQ values were only related to As and Cu; therefore, by including the missing data, the HI value would be higher. The average HQ levels of heavy metals at the county level during 2015–2021 were determined to be 0.44 for As, 0.12 for Cd, 0.15 for Hg, 0.06 for Cr, 0.03 for Pb, 0.004 for Ni, and 0.02 for Cu. Based on Fig. 5(B), the total ILCR values of the heavy metals ranged from 1.39 × 10^−4^ in Mohammadabad Zarach to 2.02 × 10^−3^ in Askarieh. The average ILCR values of As, Cd, Cr, and Pb in rural communities of Yazd County were determined to be 1.96 × 10^−4^ (significant increased cancer risk), 1.87 × 10^−4^ (significant increased cancer risk), 1.58 × 10^−5^ (probable increased cancer risk), and 7.20 × 10^−7^ (acceptable level), respectively.

The relatively high-risk values of heavy metals in drinking water in rural communities were also reported by Naddafi et al. [6]. Naddafi et al. [6] demonstrated that the occurrence of elevated HQ values (HQ > 1) for As exposure via drinking water in rural communities of Iran (8.5 %) was much higher than that in Iranian urban areas (1.5 %). They reported that the average ILCRs of exposure to As, Cd, Cr, and Pb via drinking water in Iran were respectively 1.06 × 10^−4^, 5.89 × 10^−5^, 2.05 × 10^−5^, and 3.76 × 10^−7^. The corresponding values of HQ for As, Hg, Cr, Cd, Pb, and Ni were 0.25, 0.11, 0.08, 0.01, 0.01, and 0.01, respectively. In a study by Nkitikpor and Jemerigbe [35] on hand-dug wells, the ILCR value for Cd exposure from drinking water was estimated to be 4.4 × 10^−3^ in adults. In the study by Sharma et al. [53] on heavy metal contents in groundwater, Cr exposure through drinking water from Ropar wetland, Punjab, India was found to pose significant ILCR values (0–3.26 × 10^−3^ in winter and 1.54 × 10^−3^-2.81 × 10^−3^ in summer). In a study by Malekootian et al. [54] in northwest Iran, the ILCR of exposure to As via drinking water was reported to be 9.06 × 10^−4^. In the study of Zakir et al. [55] on quality of water resources in the Jamalpur Sadar area, Bangladesh, the ILCR values of exposure to Cd, Cr, and Pb were respectively 6.91 × 10^−6^, 1.35 × 10^−7^, and 7.86 × 10^−9^.

### 3.5 Burden of diseases induced by heavy metals in drinking water

**Fig. 6:**
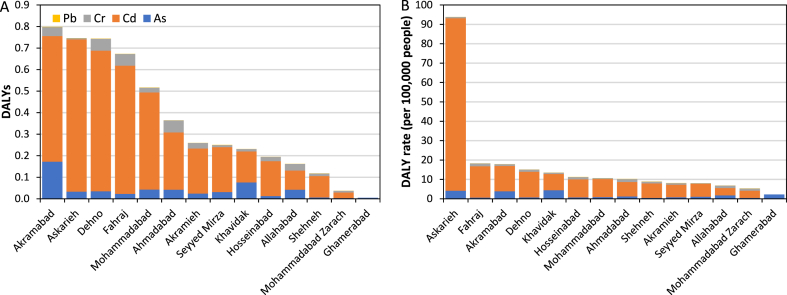
DALYs (A) nand DALY rate (B) induced by exposure to heavy metals through drinking water by risk factor and community in rural areas of Yazd County.

The burden of disease induced by exposure to heavy metals through drinking water by risk factor, cancer type, and community in rural areas of Yazd County are shown in Table 2 and Fig. 6(A and B). As indicated in Table 2 and Fig. 6(A and B), the average values of health loss indicators from exposure to heavy metals through drinking water were as follows: 0.21 for cancer incidence, 0.57 for cancer incidence rate (per 100,000 people), 9.83 × 10^−2^ for deaths, 2.68 × 10^−1^ for death rate, 4.91 for YLLs, 1.92 × 10^−1^ for YLDs, 5.10 for DALYs, 13.9 for DALY rate, and 96.2 % for share of YLLs in DALYs. The share of YLLs in DALYs varied by cancer type and rose with decreasing the cancer SR from 81.8 % for skin cancer (with the SR of 91.3 %) to 98.0 % for lung cancer (with the SR of 16.2 %). The contributions of the heavy metals to the attributable DALYs were determined to be 82.3 % for Cd, 10.7 % for As, 6.9 % for Cr, and 0.1 % for Pb. The attributable DALYs by community ranged from 4.40 × 10^−3^ in Dorbid to 0.8 in Akramabad. The three highest DALY rates by community were observed in Askarieh (93.8), Fahraj (18.2), and Akramabad (17.8) and three lowest ones were related to Allahabad (6.8), Mohammadabad Zarach (5.3), and Dorbid (2.2).

**Table 2:** Burden of disease induced by exposure to heavy metals through drinking water by risk factor and cancer type in rural communities of Yazd County.

Heavy metal Parameter	As	Cd	Cr	Pb	All the heavy metals
**Cancer type**	Skin cancer	Lung cancer	Lung cancer	Kidney cancer	All the causes
**Incidence (number of cases)**	1.03E-1	9.83E-2	8.26E-3	3.77E-4	2.10E-1
**Incidence rate (per 100 kp)**	2.80E-1	2.69E-1	2.26E-2	1.03E-3	5.73E-1
**Deaths**	8.94E-3	8.23E-2	6.92E-3	1.37E-4	9.83E-2
**Death rate**	2.44E-2	2.24E-1	1.89E-2	3.72E-4	2.68E-1
**YLL**	4.46E-1	4.11 E+0	3.46E-1	6.54E-3	4.91 E+0
**YLD**	9.90E-2	8.53E-2	7.17E-3	5.49E-4	1.92E-1
**DALYs**	5.45E-1	4.19 E+0	3.53E-1	7.09E-3	5.10 E+0
**DALY rate (per 100 kp)**	1.49 E+0	1.14 E+1	9.61E-1	1.93E-2	1.39 E+1
**Share of YLLs in DALYs, %**	81.8	98.0	98.0	92.3	96.2

100 kp: 100,000 people.

The burden of disease values obtained in this study were relatively high as compared to those from drinking water pollutants reported in the previous studies. In the study of Abtahi et al. [40], the DALYs and DALY rate (per 100,000 people) from exposure to diethyl phthalate through drinking water in Tehran, Iran were determined to be 6.385 and 0.073, respectively. Evlampidou et al. [56] evaluated the incidence and incidence rate (per 100,000 people) of bladder cancer induced by exposure to trihalomethanes (THMs) through drinking water in the European Union during 2005–2018 to be 6561 and 1.62 × 10^−3^, respectively. In the study by Naddafi et al. [6], DALYs and DALY rate (per 100,000 people) attributed to heavy metals in drinking water were estimated to be 4642 and 5.81, respectively. Abtahi et al. [57] determined the DALY rate from elevated levels of fluoride in drinking water due to dental fluorosis in Iran, 2017 to be 4.31. In the study by Dobaradaran et al. [58], the DALY rate attributable to THMs and haloacetic acids (HAAs) in drinking water in Bushehr, Iran was found to be 8.1. Based on the latest Global Burden of Disease Studies [43], the health losses from environmental risks in Iran, 2019 in terms of DALY rate were 1418 for ambient air pollution (particulate matter and ozone), 380 for lead exposure, 345 for low temperature, 118 for unsafe water source, 70 for high temperature, 32 for unsafe sanitation, 28 for no access to handwashing facility, 13 for residential radon, and 4 for household air pollution. The DALY rates of other environmental risk factors in Iran in the literature were 91 for dietary exposure to inorganic As in Iran [59], 34.50 for public overexposure to solar ultraviolet in Iran [60], and 2.07 for BTEX (Benzene, toluene, ethylbenzene, and xylene) exposure in the outdoor air in Tehran [41].

According to Global Burden of Disease Collaborative Network [43], the disease burden of skin cancer, kidney cancer, and lung cancer in Iran exhibited upward trends, so that the DALY rates of skin cancer, kidney cancer, and lung cancer in 1990 were respectively 10.27, 19.55, and 137.68 and rose to 10.54, 31.54, and 259.78, respectively in 2019.

Due to increasing trends of the relevant cancers and the relatively high health risks associated with heavy metals (especially Cd and As) in drinking water, we recommend implementing preventive interventions in the hot-spot villages (Askarieh, Fahraj, Akramabad, Dehno, Khavidak, Hosseinabad, and Mohammadabad) of Yazd County. These interventions may include replacing drinking water sources, applying heavy metal removal methods (such as membrane processes, adsorption, and ion exchange) in water treatment plants, and using household water treatment systems to mitigate adverse health effects in the rural communities. This study assessed drinking water quality based on 27 parameters and examined the health risks and burden of disease associated with heavy metal exposure through drinking water. To achieve more accurate assessments of drinking water quality, it is necessary to include additional water quality parameters, such as microbial agents, disinfection by-products, and pesticides. Additionally, reliable methods should be developed to expand the estimation of the burden of disease to other drinking water pollutants.

## 4 Conclusions

Based on the DWQI scores, the drinking water quality in rural communities of Yazd County was characterized as good for 61 %, fair for 25 %, marginal for 2 %, and poor for 12 %. The five drinking water parameters with the highest violation percentages were Mg (100 %), TH (81 %), F (42 %), Cd (18 %), and As (10 %). The DWQI and HPI indicated that the heavy metals were the most detrimental parameters in drinking water of the rural communities. In about 20 % of the rural communities, the average HI level of heavy metals was higher than the boundary limit of one, indicating a considerable risk of non-cancer outcomes. In all the communities, the total ILCR values of the heavy metals were in the category of significant increased cancer risk. The DALY rate of exposure to the heavy metals via drinking water was 13.9, which was considered relatively high as compared to that of other drinking water pollutants obtained in the previous studies. The contributions of the heavy metals to the attributable DALYs were determined to be 82.3 % for Cd, 10.7 % for As, 6.9 % for Cr, and 0.1 % for Pb. The drinking water quality improvement through decreasing Cd and As levels below the standard values can drastically reduce the attributable burden of disease and promote the public health in the rural communities. This study assessed drinking water quality based on 27 parameters and examined the health risk and burden of disease associated with heavy metal exposure through drinking water. To enhance future assessments of drinking water quality, it is recommended to include additional water quality parameters such as microbial factors, disinfection by-products, and pesticides, as well as develop methods for expanding estimation of burden of disease.

## Data availability statement

The data that support the findings of this study are available on request from the corresponding author. The data are not publicly available due to privacy or ethical restrictions.

## CRediT authorship contribution statement

**Reza Saeedi:** Writing – review & editing, Visualization, Validation, Methodology, Investigation, Data curation, Conceptualization. **Sepideh Sadeghi:** Writing – review & editing, Writing – original draft, Formal analysis, Data curation. **Mohamadreza Massoudinejad:** Writing – review & editing, Visualization, Validation, Methodology. **Maryam Oroskhan:** Writing – review & editing, Formal analysis, Data curation. **Azita Mohagheghian:** Writing – review & editing, Writing – original draft, Visualization, Validation, Investigation. **Mohamadreza Mohebbi:** Writing – review & editing, Writing – original draft, Methodology, Conceptualization. **Mehrnoosh Abtahi:** Writing – review & editing, Writing – original draft, Visualization, Validation, Resources, Methodology, Investigation, Funding acquisition, Formal analysis, Data curation, Conceptualization.

## Declaration of competing interest

The authors declare that they have no known competing financial interests or personal relationships that could have appeared to influence the work reported in this paper.
