# Supplementary material for: Assessing drinking water quality based on water quality indices, human health risk, and burden of disease attributable to heavy metals in rural communities of Yazd County, Iran, 2015–2021
Source: Heliyon. 2024 Jul 2;10(13):e33984. doi: 10.1016/j.heliyon.2024.e33984 (PMC11637201; doi:10.1016/j.heliyon.2024.e33984)
Supplement: Multimedia component 1 [file mmc1.docx]

**Supplementary Materials**

**Assessing drinking water quality based on water quality indices, human health risk, and burden of disease attributable to heavy metals in rural communities of Yazd County, Iran, 2015-2021**

Reza Saeedi^1,2^, Sepideh Sadeghi^3^, Mohamadreza Massoudinejad^3^, Maryam Oroskhan^4^, Azita Mohagheghian^5^, Mohamadreza Mohebbi^6^, Mehrnoosh Abtahi^4,7*^

^1^ Research Institute for Health Sciences and Environment, Shahid Beheshti University of Medical Sciences, Tehran, Iran

^2^ Department of Health, Safety, and Environment (HSE), School of Public Health and Safety, Shahid Beheshti University of Medical Sciences, Tehran, Iran

^3^ Department of Environmental Health Engineering, School of Public Health and Safety, Shahid Beheshti University of Medical Sciences, Tehran, Iran

^4^ MPH Department, School of Public Health and Safety, Shahid Beheshti University of Medical Sciences, Tehran, Iran

^5^ Department of Environmental Health, School of Health, Guilan University of Medical Sciences, Rasht, Iran

^6^ Department of Civil Engineering, Faculty of Engineering, University of Ottawa, Ottawa, Canada

^7^ Environmental and Occupational Hazards Control Research Center, Research Institute for Health Sciences and Environment, Shahid Beheshti University of Medical Sciences, Tehran, Iran

^*^Corresponding author. Tel: (+98 21) 22432040; Fax: (+98 21) 22432037; Email address: mehrabtahi@gmail.com (Mehrnoosh Abtahi)

**Content**

Table. S1. Name and population of Yazd County’s villages (1).

Table. S2. Standard values and weights of input water quality parameters (2, 3).

Table S3. Exposure factors and constant values for assessment of non-carcinogenic and carcinogenic health risk via drinking water in Iran, 2019.

Table S4. Duration and disability weight for different phases of cancers induced by exposure to heavy metals via drinking water.

Table. S1. Name and population of Yazd County’s villages (1).

| **Rural community** | **Population** |
| --- | --- |
| Ahmadabad | 3,572 |
| Akramabad | 4,473 |
| Akramieh | 3,210 |
| Allahabad | 2,382 |
| Askarieh | 795 |
| Dehno | 4,938 |
| Fahraj | 3,688 |
| Dorbid | 200 |
| Hosseinabad | 1,738 |
| Khavidak | 1,718 |
| Mohammadabad | 4,844 |
| Mohammadabad Zarach | 698 |
| Seyyed Mirza | 3,107 |
| Shehneh | 1,332 |

Table. S2. Standard values and weights of input water quality parameters (2, 3).

| Parameter | Unit | Standard value | Weight |
| --- | --- | --- | --- |
| Al | mg/L | 0.1 | 0.019 |
| Alk_t_ | mg/L | - | - |
| Ca | mg/L | 300 | 0.019 |
| Cl | mg/L | 250 | 0.021 |
| EC | µS/cm | - | - |
| Fe | mg/L | 0.3 | 0.023 |
| K | mg/L | - | - |
| Mg | mg/L | 30 | 0.020 |
| NH_3_ | mg/L | 1.5 | 0.013 |
| pH | - | 6.5-8.5 | 0.008 |
| PO_4_ | mg/L | - | - |
| SO_4_ | mg/L | 250 | 0.019 |
| TDS | mg/L | 1000 | 0.061 |
| TH | mg/L as CaCO_3_ | 200 | 0.017 |
| Zn | mg/L | 3 | 0.018 |
| As | mg/L | 0.01 | 0.093 |
| Cd | mg/L | 0.003 | 0.092 |
| Cr | mg/L | 0.05 | 0.092 |
| Cu | mg/L | 2 | 0.048 |
| F | mg/L | 0.5-1.5 | 0.055 |
| Hg | mg/L | 0.006 | 0.073 |
| Mn | mg/L | 0.1 | 0.044 |
| Ni | mg/L | 0.07 | 0.073 |
| NO_2_^-^ | mg/L | 3 | - |
| NO_3_^-^ | mg/L | 50 | 0.055 |
| Pb | mg/L | 0.01 | 0.092 |
| Turbidity | NTU | 1 | 0.044 |

Table S3. Exposure factors and constant values for assessment of non-carcinogenic and carcinogenic health risk via drinking water in Iran, 2019.

| **Parameters** | **Unit** | **Value/distribution** | **References** |
| --- | --- | --- | --- |
| Age | Years | 35 | Statistical Center of Iran (1) |
| Body weight (BW) | kg | 68.72 | Alikhani (4) |
| Water ingestion rate (IR) | L/d | 2 | Abtahi, Yaghmaeian (5) |
| Height | m | 164.60 | Alikhani (4) |
| Skin area (SA) | cm^2^ | 17503.1 | Calculation based on Coley and Beisteiner (6) |
| Fraction of surface skin in contact with water (F) | dimensionless | Uniform (0.65) | Dobaradaran, Shabankareh Fard (7), Zhang, Zhang (8) |
| Skin permeability constant (PC) | cm/h | 0.001 for As | USEPA (9) |
|  |  | 0.001 for Cd |  |
|  |  | 0.002 for Cr |  |
|  |  | 0.001 for Cu |  |
|  |  | 0.001 for Hg |  |
|  |  | 0.001 for Ni |  |
|  |  | 0.001 for Pb |  |
| Oral reference dose (RfD) | mg/kg.d | 0.0003 for As | USEPA (9), Ngo, Watchalayann (10), Zeng, Wei (11) |
|  |  | 0.0005 for Cd |  |
|  |  | 0.003 for Cr |  |
|  |  | 0.04 for Cu |  |
|  |  | 0.0003 for Hg |  |
|  |  | 0.02 for Ni |  |
|  |  | 0.0035 for Pb |  |
| Slope factor (SF) | 1/( mg/kg.d) | 1.5 for As | USEPA (9), Ngo, Watchalayann (10), Zeng, Wei (11) |
|  |  | 6.1 for Cd |  |
|  |  | 0.084 for Cr |  |
|  |  | 0.0085 for Pb |  |
| Exposure time (ET) for bathing | h | 0.28 | Moya, Phillips (12) |
| Exposure frequency (EF) | d/y | 365 | Abtahi, Yaghmaeian (5) |
| Exposure duration (ED) | year | 7 | Abtahi, Yaghmaeian (5) |
| Averaging time (AT) | day | 2555 | - |

Table S4. Duration and disability weight for different phases of cancers induced by exposure to heavy metals via drinking water.

| **Parameter** | **Unit** | **Skin cancer** | **Lung cancer** | **Kidney cancer** | **References** |
| --- | --- | --- | --- | --- | --- |
| Duration of diagnosis and treatment phase (*D_dt_*) | y | 0.08 | 0.5 | 0.42 | Dobaradaran, Shabankareh Fard (7); Soerjomataram, Lortet-Tieulent (13); Naddafi, Mesdaghinia (14) |
| Duration of remission to cure phase )*D_rc_*( | y | 5.00 | 6.00 | 5.00 |  |
| Duration of remission to death phase )*D_rd_*) | y | 0.33 | 0.20 | 0.90 |  |
| Duration of pre-terminal phase (*D_pt_*) | y | 0.33 | 0.20 | 0.90 |  |
| Duration of terminal phase (*D_t_*) | y | 0.33 | 0.20 | 0.90 |  |
| Disability weight of diagnosis and treatment phase (*DW_dt_*) | - | 0.26 | 0.72 | 0.27 |  |
| Disability weight of remission to cure phase )*DW_rc_*( | - | 0.19 | 0.47 | 0.18 |  |
| Disability weight of remission to death phase )*DW_rd_*) | - | 0.19 | 0.47 | 0.18 |  |
| Disability weight of pre-terminal phase (*DW_pt_*) | - | 0.81 | 0.91 | 0.64 |  |
| Disability weight of terminal phase (*DW_t_*) | - | 0.93 | 0.93 | 0.93 |  |
| Survival rate (*SR*) | - | 0.913 | 0.162 | 0.638 | Nuffield Trust (15) |

**References**

1. Statistical Center of Iran. National Population and Housing Census in 2016. Tehran: Statistical Centre of Iran (SCI); 2017.

2. Institute of Standards and Industrial Research of Iran. Drinking water - Physical and chemical specifications, No. 1053, 5th ed. Tehran: Institute of Standards and Industrial Research of Iran; 2010.

3. Abtahi M, Golchinpour N, Yaghmaeian K, Rafiee M, Jahangiri-rad M, Keyani A, et al. A modified drinking water quality index (DWQI) for assessing drinking source water quality in rural communities of Khuzestan Province, Iran. Ecological indicators. 2015;53:283-91.

4. Alikhani S. A National Profile of Noncommunicable Disease Risk Factors in the I.R. of Iran. Tehran: Ministry of Health and Medical Education; 2005.

5. Abtahi M, Yaghmaeian K, Mohebbi MR, Koulivand A, Rafiee M, Jahangiri-rad M, et al. An innovative drinking water nutritional quality index (DWNQI) for assessing drinking water contribution to intakes of dietary elements: A national and sub-national study in Iran. Ecological Indicators. 2016;60:367-76.

6. Coley DA, Beisteiner A. Carbon dioxide levels and ventilation rates in schools. International journal of ventilation. 2002;1(1):45-52.

7. Dobaradaran S, Shabankareh Fard E, Tekle-Röttering A, Keshtkar M, Karbasdehi VN, Abtahi M, et al. Age-sex specific and cause-specific health risk and burden of disease induced by exposure to trihalomethanes (THMs) and haloacetic acids (HAAs) from drinking water: An assessment in four urban communities of Bushehr Province, Iran, 2017. Environmental Research. 2020;182:109062.

8. Zhang Y, Zhang N, Niu Z. Health risk assessment of trihalomethanes mixtures from daily water-related activities via multi-pathway exposure based on PBPK model. Ecotoxicology and Environmental Safety. 2018;163:427-35.

9. USEPA. Regional Screening Levels (RSLs) - Generic Tables Washington DC: United States Environmental Protection Agency; 2021 [updated 2021. Available from: <https://www.epa.gov/risk/regional-screening-levels-rsls-generic-tables>.

10. Ngo HTT, Watchalayann P, Nguyen DB, Doan HN, Liang L. Environmental health risk assessment of heavy metal exposure among children living in an informal e-waste processing village in Viet Nam. Science of The Total Environment. 2021;763:142982.

11. Zeng F, Wei W, Li M, Huang R, Yang F, Duan Y. Heavy Metal Contamination in Rice-Producing Soils of Hunan Province, China and Potential Health Risks. International Journal of Environmental Research and Public Health. 2015;12(12):15584-93.

12. Moya J, Phillips L, Schuda L, Wood P, Diaz A, Lee R, et al. Exposure Factors Handbook. Washington DC: US Environmental Protection Agency; 2011.

13. Soerjomataram I, Lortet-Tieulent J, Ferlay J, Forman D, Mathers C, Parkin DM, et al. Estimating and validating disability-adjusted life years at the global level: a methodological framework for cancer. BMC medical research methodology. 2012;12:125.

14. Naddafi K, Mesdaghinia A, Abtahi M, Hassanvand MS, Beiki A, Shaghaghi G, et al. Assessment of burden of disease induced by exposure to heavy metals through drinking water at national and subnational levels in Iran, 2019. Environmental Research. 2022;204:112057.

15. Nuffield Trust. Cancer survival rates. London, UK: Nuffield Trust & The Health Foundation; 2021.
